# A Review of Approaches for Mitigating Effects from Variable Operational Environments on Piezoelectric Transducers for Long-Term Structural Health Monitoring

**DOI:** 10.3390/s23187979

**Published:** 2023-09-19

**Authors:** Andreas J. Brunner

**Affiliations:** Laboratory for Mechanical Systems Engineering, Empa, Swiss Federal Laboratories for Materials Science and Technology, CH-8066 Dübendorf, Switzerland; andreas.brunner@empa.ch

**Keywords:** piezoelectric transducers, structural health monitoring, long-term monitoring, variable operating conditions, maintenance and repair

## Abstract

Extending the service life of ageing infrastructure, transportation structures, and processing and manufacturing plants in an era of limited resources has spurred extensive research and development in structural health monitoring systems and their integration. Even though piezoelectric transducers are not the only sensor technology for SHM, they are widely used for data acquisition from, e.g., wave-based or vibrational non-destructive test methods such as ultrasonic guided waves, acoustic emission, electromechanical impedance, vibration monitoring or modal analysis, but also provide electric power via local energy harvesting for equipment operation. Operational environments include mechanical loads, e.g., stress induced deformations and vibrations, but also stochastic events, such as impact of foreign objects, temperature and humidity changes (e.g., daily and seasonal or process-dependent), and electromagnetic interference. All operator actions, correct or erroneous, as well as unintentional interference by unauthorized people, vandalism, or even cyber-attacks, may affect the performance of the transducers. In nuclear power plants, as well as in aerospace, structures and health monitoring systems are exposed to high-energy electromagnetic or particle radiation or (micro-)meteorite impact. Even if environmental effects are not detrimental for the transducers, they may induce large amounts of non-relevant signals, i.e., coming from sources not related to changes in structural integrity. Selected issues discussed comprise the durability of piezoelectric transducers, and of their coupling and mounting, but also detection and elimination of non-relevant signals and signal de-noising. For long-term service, developing concepts for maintenance and repair, or designing robust or redundant SHM systems, are of importance for the reliable long-term operation of transducers for structural health monitoring.

## 1. Introduction

**Figure 1:**
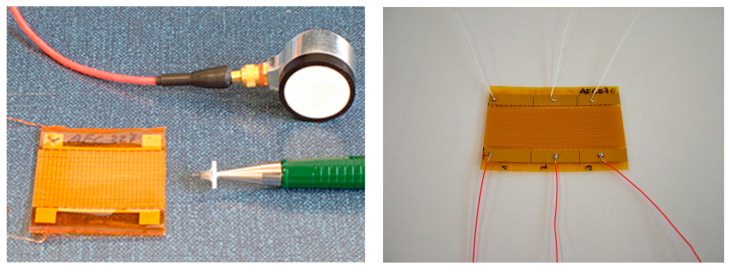
Photos from the authors’ laboratory: (**left**) AFC transducer with actuating and sensing capability manufactured according to the design described by [19] compared with a commercial 150 kHz resonant acoustic emissions sensor, (**right**) AFC with three independently controllable electrode segments on one device.

Structural Health Monitoring (SHM) [1] is roughly the periodic or continuous application of technical methods implemented in a structure or structural element with the aim to assess its integrity, fitness for use, remaining service-life under specified operating conditions, or to optimize the maintenance required for this. In an era of limited resources, SHM is gaining in importance for extending the service life of ageing infrastructure and transportation structures. Standardization of SHM applications in guidelines or test procedures has been rather slow, but recently is increasing, especially in the construction industry, see, e.g., [2]. A specific type of SHM is Condition Monitoring (CM) of machinery for which several standard guidelines have been developed, see, e.g., [3,4,5,6,7]. There are different sensor technologies for acquiring SHM data, see, e.g., [8,9,10]. For structural wave or vibration based SHM, piezoelectric transducers and fiber optics are widely investigated and implemented [11,12]. For piezoelectric transducers, PZT (lead-zirconate-titanate) is one of the main transducer materials, see, e.g., [13]. The integration of SHM transducers of any kind into the monitored objects is a challenge, and requires careful design of the SHM system. Integration of piezoelectric transducers into structures may benefit from special types of planar, thin transducers. These comprise so-called Active and Macro Fiber Composites (AFC and MFC, respectively) that are flexible [14,15] (see Figure 1), piezoelectric patches [16], piezoelectric wafer active sensors (PWAS) [17,18] or piezoelectric disks that are commercially available.

**Figure 2:**
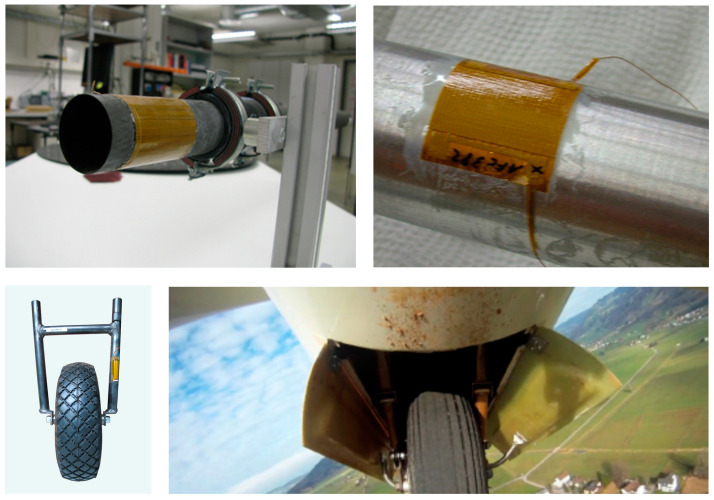
Examples of flexible AFC or MFC mounted directly on curved surfaces without waveguides from the authors’ laboratory, (**top left**) AFC mounted on a CFRP strut for compensation of thermal expansion, (**top right**) AFC mounted on an aluminum pipe for leak monitoring [35], (**bottom**) MFC mounted on a glider plane landing wheel gear for electro-mechanical impedance (EMI) monitoring [36].

The durability of such devices has been investigated and discussed by, e.g., [18,20]. Mechanical strains of the order of typical engineering strains (up to about 0.25%) or impact of foreign objects may be detrimental to the performance of piezoelectric transducers. Hence, various “packaging” approaches for AFC, MFC, or PWAS may improve their durability when integrated into structures. Advantages and disadvantages of different embedding methods (Figure 2) are discussed in detail by, e.g., [11,12].

Depending on the application, performing SHM with different sensor types, often with complementary measurement principles, is advantageous, see, e.g., [21]. Data fusion for the analysis of signals from different transducer types reviewed, e.g., by [22], looks promising for future SHM. Artificial intelligence is also playing an increasingly important role in the related signal and data analysis, see, e.g., [23,24,25].

Effects from different operational environment on piezoelectric transducers are reviewed by the following papers: buildings by [26], vibrational condition monitoring by [27], wind turbines by [28], railway vehicles by [29], hydrogen pressure vessels by [30], or structures in low earth orbits by [31]. The aspects of long-term operation of SHM system and transducer durability, however, has, so far, only received scant attention at best [32]. Variable operating environments lead to different damage mechanisms in the components of the SHM system, i.e., the transducers, signal transmission, data acquisition, data storage and analysis, often acting on different time scales. Identifying potential synergistic effects, both positive or negative for the service life of SHM systems, can hence be quite challenging.

Depending on the demand for specific types of transducers, e.g., some Acoustic Emission sensors made in low numbers (as shown by their serial numbers), manufacturing may be still mostly manual rather than fully or partly automated [33,34]. Besides the manufacturing processes, the quality of the piezoelectric material is another important factor. Overall, both may result in some variability in transducer properties that, in principle, could affect their long-term durability. However, to the best knowledge of the author, no information on such effects exists in the public domain.

This review consists of four parts: Section 2 summarizes different operational environments and identifies critical influences for piezoelectric transducers. In Section 3, a short review summarizes published experience with piezoelectric transducers for long-term SHM. Section 4 first presents effects from ambient climate (temperature and humidity) and from mechanical loads and the respective mitigation approaches in detail, complemented by a discussion of selected special operational environments. Section 5 briefly summarizes the main aspects and adds a brief outlook. Section 6 then provides conclusions.

## 2. Relevant Operational Environments

Operational environments for piezoelectric transducers comprise a variety of service conditions. Primary factors determining the service life are (1) ambient temperature, elevated or low and either roughly constant or variable, and (2) mechanical loads, either constant, variable (e.g., structural vibrations) or short-term (e.g., impact of foreign objects) or variations in ambient pressure that act on the objects to which the transducers are coupled and/or to the transducers and their mounting devices directly. Secondary factors are (3) ambient humidity, both in gaseous and liquid form, (4) other chemical species present as fluids or gases in the operating environment, e.g., seawater or oxidizing media, (5) alternating electromagnetic fields with different frequencies (energies), (6) particle radiation, and, of course, (7) combinations of these factors.

**Figure 3:**
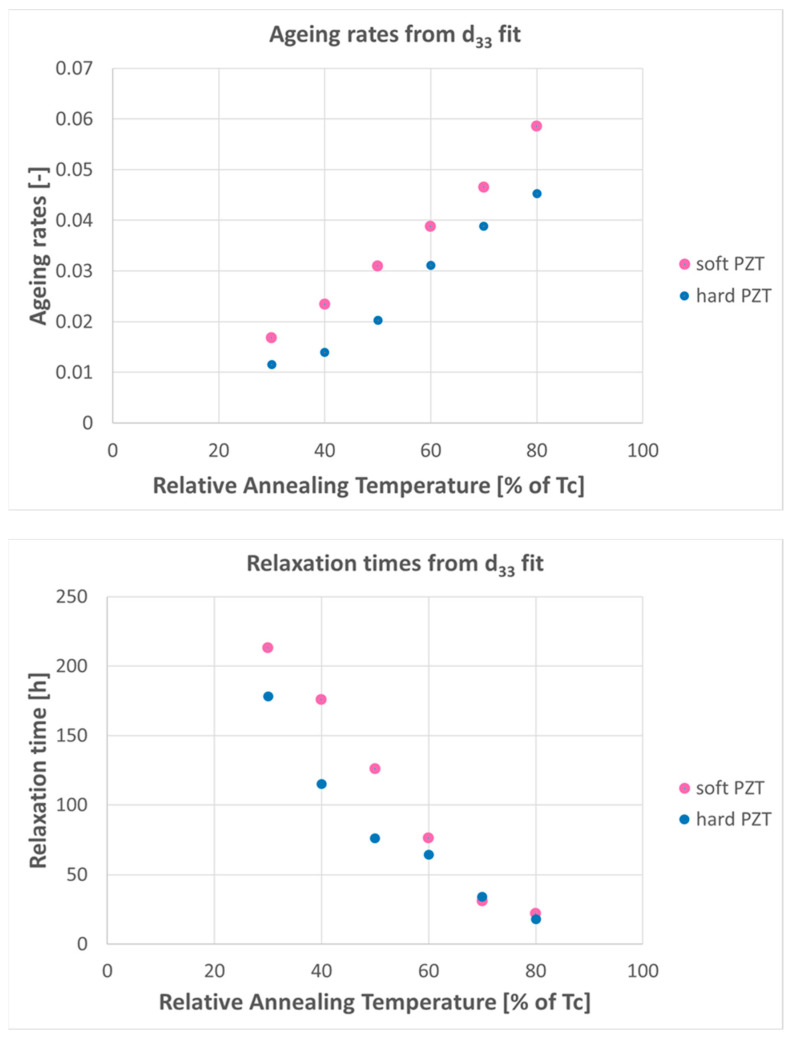
Thermal ageing effects from 10 min annealing on piezoelectric PZT material, graphical presentation of data in tables 1 (**top**) and 2 (**bottom**) in [37], indicating increased ageing rates and decreasing relaxation times with increasing annealing temperatures, respectively.

Variable temperature environments can affect piezoelectric transducers in different ways. Elevated temperatures and large temperature variations may change the polarization of piezoelectric materials. The Curie-temperature (T_C_) is the upper temperature limit for operating piezoelectric transducers, but in practice, a significantly lower temperature limit is necessary for long-term operation. Short, ten minute annealing at temperatures between 30% and 80% of the Curie-temperature [37] on two types of piezo-material samples (one “soft”, one “hard”, i.e., with higher and lower domain wall mobility, respectively [38]), yielded clear indications of performance degradation for both types. The d_33_ piezoelectric charge coefficient showed an increasing drop in the value with increasing annealing temperature compared with material before annealing. The drop was more significant for soft than for hard piezoelectric materials. This behavior was interpreted as an increasing ageing effect of the piezoelectric charge constant d_33_, i.e., the polarization in a piezoelectric material generated per unit of mechanical stress applied parallel to the polarization direction. Simultaneously, relaxation times for the polarization decreased with increasing annealing temperature as well (Figure 3).

In operational environments without climate control, there are daily and seasonal temperature and humidity variations. For aircrafts, as an example, operational temperatures vary between about −50 °C and +70 °C within a few hours, and the respective relative humidity ranges between a few and 100 percent. In space, operational temperatures do vary even more, but there are no humidity effects. On the one hand, temperature variations may yield thermal stresses in the transducers from differences in the coefficient of thermal expansion leading to fatigue damage, where specifications for commercial transducers define the allowable operating temperature range in order to mitigate this; and on the other hand, temperature variations may directly affect the sensitivity of the PZT-element. There are two mechanisms. The first is temperature-induced depolarization of the PZT resulting in lower sensitivity. The second mechanism is pyroelectricity, inducing spurious signals in the PZT-elements directly [13]. Pyroelectricity usually does not degrade the transducer performance, but non-relevant signals in the SHM data may yield “big” data sets requiring higher computational power and data storage and more elaborate analysis.

Variable mechanical loads may cover a broad range of frequencies and amplitudes. Load types can be tensile, compressive, shear, or combinations. Impact loads, high acceleration or deceleration outside specified operating limits or test object failure can also induce damage in the transducers. Examples of impact loads by foreign objects are hail, bird strikes, falling rocks or trees or tool dropping. Improper set-up or operation, e.g., causing collisions with vehicles or other moving structures, is also feasible. Depending on the location of the SHM system, unintentional contact by people or vandalism may also result in damage, e.g., in the SHM of civil engineering structures accessible to the public.

There are special operating environments, among them, nuclear installations (e.g., nuclear power plants or particle accelerators for research or health treatments), structures in space, and processing facilities handling potentially explosive materials, or yielding such materials as products or waste. The latter comprise, e.g., oil or gas production facilities and refineries, chemical production plants, and processes yielding large amounts of fine grained powders or dust particles (e.g., mills with grinding or abrasion processes). Fire is also a potential hazard in many of these facilities. Underground installations or spaces, e.g., tunnels or mines, often hold mechanical loads, temperature and humidity roughly constant, but may yield damage to SHM transducers and systems by exposure to corrosive media. Earthquake prone locations require special attention if the SHM system is able to monitor structures during or after seismic events of a specified magnitude [39,40].

## 3. Experience Published from Long-Term Monitoring with Piezoelectric Transducers

A comprehensive review recently compiled published experience from long-term SHM with piezoelectric transducers [32]. With “long-term” defined loosely as continuous monitoring periods of about half a year or more, a search was made for quantitative service life data of the components of the SHM system. The main conclusion of this review was that only scant quantitative data on the performance of SHM systems and piezoelectric transducers is publicly available so far. Some SHM service providers have likely accumulated experience on damage and failure of transducers and other components in various operational environments. However, even if documented, failure listings are usually not published. One exception is a bridge monitoring report on Acoustic Emission [41], which lists all component damage and failures in detail. A questionnaire distributed within the Committee on Acoustic Emission of the German Society for Nondestructive Testing in 2021, see [32] for detailed discussion, yielded further information. The limited number of responses received so far (six) may be too small for statistically relevant conclusions, but they provide, nevertheless, valuable indications of critical issues.

**Figure 4:**
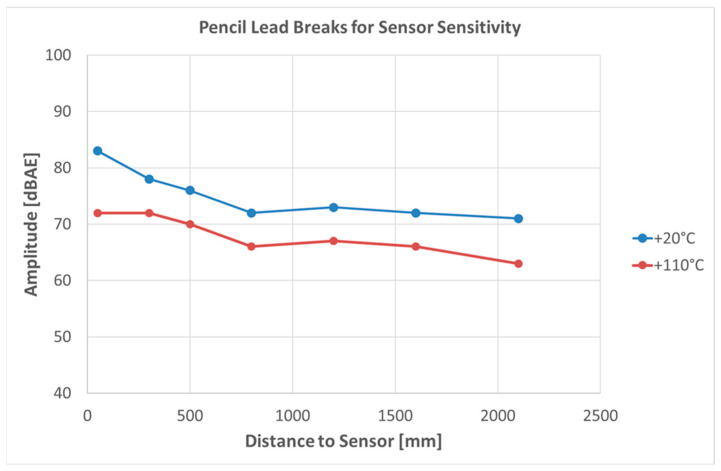
Thermal effects on PZT transducers mounted on a steel plate from lead-pencil breaks discussed in [32] (data courtesy of M. Löhr).

One issue identified in the questionnaire is the significantly reduced sensitivity of PZT-transducers operated at an elevated temperature (around +110 °C), but still within the specified operating range for the sensor type. The observation came from a short-term preliminary experiment for defining the maximum allowable sensor distance (Figure 4). Another case of observed transducer failure was tearing signal transmission cables or mounting devices, likely due to unintentional contact by third party people. This indicates that transducer mounting and signal transmission require appropriate design and installation. Controlling and limiting access to the SHM system, if feasible, is likely the best mitigation approach. Other damage and failures reported in the questionnaire consisted of several cases of component failure, either in the data acquisition, data storage or data analysis equipment (e.g., computer screen failure, power source failure), sometimes leading to a loss of data. It is hence recommended to set-up data acquisition, data storage, and analysis equipment for SHM performed outdoors in a cabin or container providing protection against environmental effects and limiting access to authorized personnel, as discussed, e.g., by [42].

After the publication of the review [32], additional examples of long-term SHM were found, see, e.g., [43,44,45]. Examples of commercial bridge monitoring in Germany, running up to six years now (Thalaubachtal Bridge since 2017) are noted in a summary presentation (in German) at a technical meeting [46]. The listing also includes a project planned from 2022–2030 with 248 Acoustic Emission sensors monitoring a highway tunnel in Munich (Germany). The continuous SHM of bridges is likely the fastest growing monitoring application. However, it may be limited by resources, both with respect to the availability of measurement systems and technical personnel for installation and maintenance, but also by a potential lack of civil engineers with sufficient know-how for interpretation of the various SHM data. No problems were reported for the monitoring projects in [46] of the SHM system induced by the operating environment. However, this does not necessarily mean that none occurred. Publishing documented company internal problem reports on damage or failure of all components of SHM systems, as well as systematically collecting such data during operation of current SHM projects, would be essential for improving the performance of future SHM systems.

## 4. Mitigation of Effects from Variable Service Environments

This section discusses mitigation of the major ambient conditions affecting the performance of piezoelectric transducers in the long-term, i.e., temperature variations and mechanical loads. Before discussing the different environments, two general mitigation procedures, namely preventive and predictive maintenance that apply to a wide range of operational environments, are worth mentioning. Predictive maintenance is a subset of preventive maintenance, for which data from the SHM systems’ performance enable predicting the optimal time for maintenance action. Signal-to-noise ratio and “noise” signals from non-relevant sources are general aspects in all applications of SHM and, hence, are discussed here as well. Selected examples of other environments with specific conditions and combinations are electromagnetic interference, nuclear radiation exposure (in nuclear facilities and in space), potentially explosive environments (oil, gas and chemical industries, and food and wood processing plants), underground facilities (mines and tunnels) and, finally, perspectives for piezoelectric energy harvesting.

### 4.1. Preventive and Predictive Maintenance

Preventive maintenance without a predictive tool is practiced by one company answering the questionnaire of the German Society for Nondestructive Testing [32]. Transducers (piezoelectric and others) and certain components of the measurement chain are replaced regularly after a service duration defined by experience (if possible from the respective operational environment), but are essentially independent of their effective remaining service life. This has the advantage that availability of human resources and of material or components for maintenance is projectable. Further, the schedule is adaptable to the production or operating cycles of the clients’ organization. During the down-time, software updates can be installed and a full operational check of the SHM system can be performed. This guarantees a high technical availability of the SHM system and minimizes the probability of unexpected failures at a comparatively lower cost. The definition of the maintenance intervals, of course, requires experience with the specific ambient conditions, in order to minimize the related hardware and personnel cost. Important aspects for this are the accessibility to the monitoring site and modularity of the SHM system. These do have implications for designing the SHM system and planning the client specific set-up, see, e.g., [32]. Nuclear installations, discussed in more detail below, are one example where preventive maintenance may make sense.

Predictive maintenance, see, e.g., reviews by [47,48,49], as a special case of preventive maintenance, also aims at achieving a high technical availability of the objects monitored by SHM. There are several approaches for predicting the time at which equipment maintenance is “best” performed. “Best” is typically an optimization criterion based on technical and/or cost considerations. Predictive models and, more recently, digital twins, also in combination with artificial intelligence, play increasingly important roles in this, see, e.g., [49,50,51]. The effort for implementing predictive maintenance approaches is higher than that for preventive maintenance, since the SHM data evaluation includes continuous or periodic comparison with defined limit parameters, predictive models, or digital twins. Such limits, models and digital twins have to be developed, validated, implemented, periodically assessed for their performance, and adapted, if necessary. There is copious literature on this topic. Predictive maintenance is a highly promising and possibly the most effective mitigation process for avoiding problems induced by the operational environment during long-term SHM.

### 4.2. Signal-to-Noise-Ratio and Signals from Non-Relevant Sources

Spurious signals from pyroelectricity induced by temperature variations are one example of signals from non-relevant sources. In any case, it is necessary to identify signals coming from sources not related to degradation and damage in the monitored structures. Once identified, removing them from the data set is desirable, either during acquisition or later in the signal analysis. Preferably, this shall be a first step in the analysis or even implemented as so-called front-end filter in the data acquisition, in order to reduce the computational effort in subsequent signal analysis. However, this only works if the non-relevant signals are identified unambiguously and clearly separated from the relevant signals, indicating changes in the monitored objects. Increasing the computational power handling of large signal acquisition rates may soon allow for sufficiently fast and reliable analysis, eliminating non-relevant signals on-line during acquisition.

Signal noise reduction, i.e., improving the signal-to-noise ratio if there is either stochastic or continuous noise present is different from the identification and elimination of the discrete, so-called burst-type signals coming from non-relevant sources, see, e.g., [52,53]. Sufficient signal-to-noise ratio is essential, hence, noise sources have to be identified and eliminated, or at least mitigated to acceptable levels. Signal noise reduction is discussed in many publications, see, e.g., [54,55,56]. Such procedures can be implemented into the data analysis. However, whenever feasible, elimination of the noise sources is always the best mitigation approach.

### 4.3. Temperature Effects

#### 4.3.1. Temperature and Its Variation

**Figure 5:**
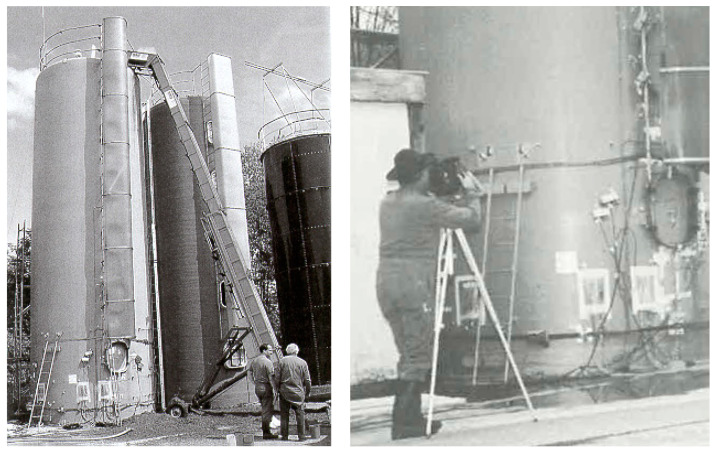
SHM field-test with acoustic emission near the manholes of a GFRP agricultural silo (photos from an advertisement flyer of the former Polymer Composites Laboratory at Empa); daily variation of exposure to sunlight yields height differences between sun exposed and shaded side of about 10 mm.

The variation of temperature may induce thermal stresses in materials and components as discussed above. Hence, both the transducers and the objects monitored may show effects from that. Figure 5 shows an example of a field test with acoustic emission on an agricultural silo made from GFRP. With a height of about 10 m, the daily difference of the thermal expansion in height between the side heated by sunlight and the back side shielded from direct exposure to the sun amounted to about 10 mm (measurements were performed in Spring and early Summer). Depending on the size of the monitored object and the variation in operating temperature, such differences may affect the sensitivity of the transducer array, especially for highly damping materials. Indeed, the respective movements may affect the mounting devices and the coupling of the transducers. For transducers, the mechanical stresses induced by temperature variations within their specified operating temperature range and possibly specified rates of temperature change are likely tolerable, leading to “normal” ageing. Effects of temperature on the piezoelectric material, however, are not limited to mechanical stresses. Electric effects, as noted above, may reduce the performance of the transducers and require mitigation if the performance reduction is significant.

#### 4.3.2. Mitigation of Temperature Effects on Piezoelectric Transducers

Higher operating temperature or temperature variations of the monitored object or its ambient reducing transducer sensitivity require lower distances between transducers if mounted in an array on structures, such as those noted above.

The basic mitigation approaches against temperature-induced effects are (1) removing the PZT-transducers from the elevated or variable temperature environment, or (2) implementing piezoelectric transducers with improved temperature-tolerant design, specifically with piezoelectric materials with a higher Curie-temperature.

For monitoring objects operated at elevated temperatures, where surface temperatures exceed the specified transducer operating range or would induce rapid performance degradation, the approach used most often are so-called waveguides. Often, these are rod-like metal components with contact plates at both ends, one fitting the structure, the other the transducer. Depending on their shape, waveguides may act as a kind of filter for certain wave-modes. A modelling study [57] explored different shapes, sizes and materials for waveguides and included temperature effects as well. One conclusion was that temperature effects, at least for the range of waveguides investigated, were not significant. The model is useful for simplifying the selection waveguide shapes and materials.

Piezoelectric materials for transducers with higher T_C_ less affected by elevated temperature environments have been reviewed by, e.g., [58,59]. A disadvantage of these materials is often lower performance compared with PZT. Availability of commercial transducers made with alternative sensing materials may be limited, however, government regulations banning the use of lead in PZT-transducers [32] may initiate the wide-spread development of alternative piezoelectric materials in the near future.

Piezoelectric transducers for low-temperature applications have received scant attention so far. Some commercial transducers, specified for operation at low temperatures, perform even down to liquid nitrogen at 77 K, or liquid helium at 4 K. However, there is no long-term experience published yet. In [60], the results after 20 cooling cycles showed no significant change in sensitivity within the experimental scatter. Until Spring of 2023, the sensor had accumulated roughly 100 cycles [61].

Theoretically, approaches for implementing transducers protected in some type of thermally shielded box, or even with active climate control with a suitable contact surface acting as thermal barrier, in principle, seem feasible. However, to the best knowledge of the author, such devices are not reported nor discussed in the literature. Possibly, the development effort and cost for such a device, as well as its operation, are prohibitive compared with alternative solutions.

#### 4.3.3. Alternative Approaches without Piezoelectric Transducers

Of course, mitigation of temperature effects on piezoelectric transducers is also feasible by implementing transducers based on alternative measurement methods. Optical fiber-based SHM systems are less sensitive to temperature and insensitive to electromagnetic interference, see, e.g., [62]. Optical fiber-based transducers for Acoustic Emission at elevated temperatures have been developed, e.g., for measurements of metal corrosion processes by [63,64]. Non-contact measurements for SHM are also an option, see, e.g., [65,66,67,68]. Laser-based methods, e.g., scanning laser vibrometry, and of “structured light”, e.g., DIC, Shearography, or thermography or laser ultrasound excitation and measurement for non-contact methods, are available for many SHM applications. Non-contact optical methods may require specific surface qualities, e.g., limited roughness and sufficiently constant reflectivity, see, e.g., [69], or pose limits on shape, e.g., no “sharp” edges/corners or no complex curved shapes inhibiting full view. Therefore, which method may provide the “best” solution for SHM requires careful consideration.

### 4.4. Mechanical Loads

Mechanical loads may act on the transducers and their mounting devices directly or on the test objects, which then transmit the loading to the transducers. Such loads may also act on other components of the measurement chain and induce damage or failure, e.g., in signal and power transmission lines, in data acquisition and data storage equipment, or in power production or supply components.

In the survey of the German Committee on Acoustic Emission, the transducers seem less affected than the other parts of the measurement chain [32]. Mitigating the impact effects of foreign objects on transducers and transducer mounting devices is feasible by choosing transducer locations that are less likely hit or by suitable mounting devices protecting the transducers. Transducer holders with permanent magnets provide constant contact pressure on magnetic test objects, e.g., steel pressure vessels, but may yield lower forces at elevated temperatures [32]. Another question is the choice of coupling agent between transducer and text object surface; mounting with an adhesive seems to be the preferred option for long-term SHM depending on the test object, e.g., conical transducers mounted without any coupling agent avoided ageing effects in the coupling medium [32].

Special cases of mechanical loads are coming from natural hazards, e.g., earthquakes, hurricanes, tsunamis, landslides, flooding, or falling rocks. Earthquakes, depending on their magnitude, are either hardly felt or can cause anything between insignificant damage and large-scale collapse of infrastructure. Debris from failing infrastructure or the ambient caused by any of the above hazards can hit monitored objects and SHM systems, even if the test objects themselves remain essentially intact. Critical infrastructure resilience in general, as discussed in [70], comprises aspects of robustness, redundancy, resourcefulness and rapidity. The complexity and interdependency of modern infrastructure [40] makes mitigating damage from natural hazards difficult. Besides the damage due to mechanical loads, the SHM system operation may be affected by power failures, interruption of signal lines or telecommunication problems.

**Figure 6:**
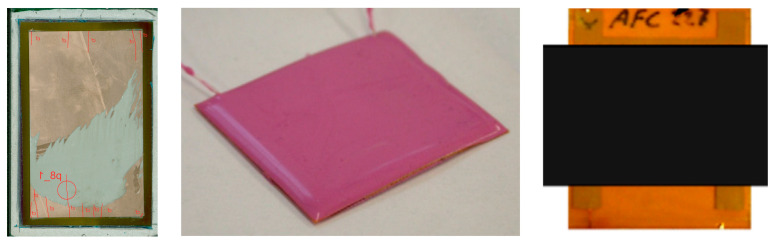
Packaging approaches for piezoelectric wafers and AFC: (**left**) wafer packaged in plain or pre-stressed thin polymer films (showing the specimen label and cracks labelled by “b” in red) [71]; (**middle**) AFC packaged in silicone rubber; (**right**) AFC packaged between pre-stressed carbon fiber laminates [72].

For embedded piezoelectric transducers, such as AFC, MFC, PWAS, different “packaging” of such devices (Figure 6) has yielded improved performance, both with respect to quasi-static and cyclic fatigue loads with strains (e.g., typical engineering strains up to about 0.25%) exceeding the failure strain of the piezoelectric materials (clearly below 0.2%) [71,72]. However, not all approaches worked as expected, e.g., silicon rubber packaging resulted in uneven thickness and in high attenuation, since no sensor response was observed during testing. However, long-term SHM applications with such devices, to the best knowledge of the author, have not been reported yet. On the one hand, embedding transducers inside structures provides some protection against effects from the operating environment, but on the other hand, may make replacement or repair more difficult. Mounting transducers on easily accessible surfaces and protecting them by suitable covers may be preferable. However, as noted in [73], the cover or the mounting devices shall not interfere with signal propagation paths or, in general, reduce transducer sensitivity below acceptable levels.

Pressure waves propagating in air may induce structural waves in the monitored objects and hence in the measurement chain. Even if this does not cause damage to the transducers and the measurement chain, it may yield large amounts of non-relevant signals in the data files. Such air-coupled ultrasound [74,75] may come from many different ambient sources. Examples are dropping objects, e.g., tools, traffic noise, operations or processes nearby creating audible and ultrasound noise. Even clapping hands close to the setup excited signals in the piezoelectric transducers, as observed by the author. Of course, this only happens if the source mechanisms produce waves of sufficient intensity with frequency content above the high-pass frequency threshold of the preamplifier or of the data acquisition channel. Hence, running the data acquisition and recording typical ambient noise and non-relevant signals before starting the SHM is essential. Such data then allow for defining the threshold and/or the frequency filter range. This may mitigate the problem to some extent, but will not successfully eliminate all such signals in all cases. A disadvantage of such front-end filters is the potential loss of “real” damage signals with characteristics similar to signal noise or signals from non-relevant sources. Service environments with large variations in ambient noise or slowly changing with time during long-term SHM may require periodic analysis for the identification of changes in signal noise or in sources of non-relevant signals. Analogous to mitigating temperature effects discussed above, considering non-contact SHM can also mitigate the effects from mechanical loads acting on the test objects.

### 4.5. Electromagnetic Interference

Many fracture phenomena in materials are known to yield short-term electromagnetic emissions [76,77]. Short term variable electromagnetic fields present in many SHM environments (e.g., from turning on electric power for heavy machinery or lights) usually do not induce damage in the piezoelectric transducers [17,18]. Such fields, however, may yield spurious, non-relevant signals in piezoelectric transducers. Full electromagnetic shielding of the transducers and of the signal transmission lines is difficult, especially for high-frequency fields (MHz to GHz). Identifying such signals prior to testing and eliminating them by front-end filters or in the data analysis is likely the best approach, see, e.g., [54,56].

Electrostatic discharges with the potential to damage electronic chips are likely more relevant for data acquisition, data storage and computers than for the transducer. A potential exception may be transducers with integrated preamplifiers. Measures for preventing loss of data or of test control by such mechanisms, but also reliability of electronic components and equipment, in general, are discussed, e.g., in [78].

Lightning striking the monitored object directly or objects nearby is potentially detrimental, but to the best of the authors’ knowledge, such cases are not found in the published literature.

Electro-magnetic interference or discharges may also affect wireless electronic signal transmission devices. Reliability assessment comparing the long-term performance of cable-based versus wireless signal transmission is necessary for deciding which of the two is better suited for a specific SHM application. Depending on the power consumption of the SHM system and the storage capacity, respectively, it may be advisable to combine the batteries of wireless devices with local energy harvesting devices. Energy harvesting modules based on piezoelectric elements for power generation are discussed in more detail below. Of course, other types of energy sources may be necessary depending on the power consumption requirements of the SHM system or the specific application.

### 4.6. Selected Special Operational Environments and Combinations

#### 4.6.1. Nuclear Facilities

The best-known examples are nuclear power plants; others are nuclear or particle research facilities (e.g., accelerators), or medical radiation therapy equipment. The major limitation for mitigation measures performed by personnel, such as repair or replacement, is due to radiation safety regulations. Depending on the radiation levels, access during operation may be prohibited for humans or at best feasible for short time intervals. The use of robots for maintenance and monitoring is feasible [79,80,81], but may also be limited in high-nuclear radiation environments, depending on the design of the electronic robot control.

In [18], high-temperature and nuclear radiation effects on PWAS are reviewed, and the conclusion is that no significant changes in the microstructure of the PZT material were found after both exposure types. However, temperature effects occurred in frequency, e.g., in resonance and anti-resonance frequencies. Nevertheless, piezoelectric transducers looked suitable for SHM in harsh, high-temperature and nuclear radiation environments. Nevertheless, other research reports depolarization effects and sensitivity reductions in piezoelectric materials after various irradiation exposures, see, e.g., [82,83,84]. Likely, the SHM of nuclear facilities will benefit from preventive or, if sufficiently developed, predictive maintenance. Essentially, this is due to the limited access during operation, with scheduled shut-offs as only option for access to the SHM system for maintenance.

#### 4.6.2. Potentially Explosive Operating Environments

A special and partially regulated environment are facilities or processes that produce gases or small-size particles (e.g., dust, powders) with the potential to explode under certain ambient conditions. Regulations by the European Union (EU) go by the designation of “ATEX” (from the French “Atmosphères Explosives”) or on the international level by “IECEX System” from (“International Electrotechnical Commission System for Certification to Standards Relating to Equipment for Use in Explosive Atmospheres”). Examples are oil and gas exploration, production, transport and refining processes [85,86], chemical plants [87,88], but also sugar production plants [89,90] and wood-processing facilities [91,92].

ATEX documents provide a classification of potentially explosive environments and specify the requirements for measurement technology deployed and operated in such environments. IECEX certificates confirm conformance with the respective requirements. However, these are not mandatory per se, different from ATEX requirements within the EU. Commercial manufacturers offer transducers, signal transmission and data acquisition equipment conforming to these regulations, see, e.g., [93,94,95]. Implementation of such equipment for SHM in potentially explosive environments will essentially eliminate the risk of explosions induced by the SHM system. Of course, this does not necessarily mitigate all damage to the transducers or the SHM system by explosions or fires caused by other mechanisms.

#### 4.6.3. Space Applications

Space, as an operating environment, comprises different ranges. There are so-called Low Earth Orbits (LEO), defined as altitudes below 1000 km with a lower limit around 160 km [31]. Further, geosynchronous orbits (GEO, around 36,000 km) are between medium and high earth orbit (MEO and HEO); all subject to different operating environments [96]. Deep Space, on the other hand, defined by NASA [97], extends beyond our moon or at least two million kilometers from earth by the International Telecommunication Union [98].

According to [31] the LEO, the space environment consists of vacuum with elemental and molecular gases or plasmas, ultraviolet and ionizing radiation, electro-magnetic fields, solar flux, and (micro-)meteoroids. In [96], the authors state (cite) “*The dominant environmental components and their effects on spacecraft in different orbits, i.e., the geosynchronous orbit (GEO), the low earth orbit (LEO), the medium earth orbit (MEO), and the high earth orbit (HEO), are investigated, respectively. The space environment that should be taken into particular consideration is summed up to facilitate the design of the spacecraft in a specific orbit. It can be seen that various space environmental components have different impacts on the spacecraft operation, which could lead to numerous anomalies. It is noticeable that the specific environment analysis for different orbits is the very demanding basis of spacecraft maintenance*”.

The complexity and variability of the space environment and the interaction between environmental factors make modelling potential effects on satellites and SHM systems extremely difficult, even if data for specific environmental parameters, such as temperature or irradiation, are available from experiments under controlled laboratory conditions. In [31], the authors emphasize (cite) “*… the necessity of a thorough understanding of the space environment for spacecraft designers and engineering performance issues that may arise from space environment exposure to materials. Flight experiments from the European Space Agency (ESA), Japan Aerospace Exploration Agency (JAXA), and the National Aeronautics and Space Administration (NASA) have been presented that focus on space environmental exposure of materials in order to design and operate future space systems successfully. In designing spacecraft, we should reflect on the results from these experiments and emphasize the importance of continuing to accumulate long-term measurement data of the space environment and its effects*”.

Access to space structures for in-service maintenance or repair is even more limited than in nuclear facilities and hence costly. Spacecrafts in LEO were serviced or repaired during several space missions [99], but repair for satellites at higher altitudes is not yet feasible. Repairable spacecraft designs are under development [100], but so far, damage essentially has to be limited at the design stage. This implies a sufficiently robust design of all relevant components in order to achieve the expected service-life. A prime example of robust design is a two Voyager spacecraft launched by NASA in 1977, which achieved the longest operation time of any deep space mission so far, reaching the edge of our solar system after 45 years in 2022 [101]. How simpler and less-costly repairability, once available, will affect satellite design, and hence, SHM, in space in the future, remains to be seen.

### 4.7. Mines and Other Underground Facilities

Salt mines are potential long-term storage for radioactive waste from nuclear power plants and other sources. They provide examples of long-term monitoring over many years [32,102]. Information on problems with transducers and other components of the SHM systems are scarce. The environment is typically rather dry at roughly constant temperature. Observed failures caused by corrosive media damaged the preamplifiers. Mitigation includes use of corrosion-resistant materials as far as possible and sealing of all connections.

Approaches and issues for monitoring of car or railway tunnels in [103] discuss experiments investigating the response of acoustic emission and vibration monitoring data during rock block collapse in the tunnel. Conclusions are (cite) “*In order to apply the AE-Vibration joint monitoring in practical tunnel engineering, it is necessary to find the key blocks using detection equipment, which can be realized through existing technology and block theory. This study proposes to replace low-frequency microseismic sensors with high-frequency acoustic emission sensors to obtain signals from key blocks in the range of 5–10 m.*”. With respect to signals from non-relevant sources and alternative measurement methods, the authors note (cite) “*The AE sensor must be installed above a certain height over the ground to effectively shield the noise signals from human activities and vehicles, which requires preparation before monitoring. Natural frequency monitoring can be performed by mounting a three-component wireless acceleration sensor on the surface of the key block or using a non-contact laser vibrometer for rapid testing. The latter is more convenient and can be operated at a certain distance*”. The publication, however, does not mention problems relating to long-term SHM.

### 4.8. Piezoelectric Energy Harvesting in Various Oprating Environments

Piezoelectric devices, besides the transducer function for SHM, can also operate as an energy harvester [12,104,105]. For providing electric power to SHM systems or wire-less transducer nodes, piezoelectric energy harvesters compete with other energy sources. A review [104] notes various energy sources, including photovoltaic, thermoelectric, piezoelectric, and radio frequency. Another review [106] on energy harvesters for railway applications notes multiple energy sources, including vibration, wind, solar, thermal, magnetic field and acoustic energy. The authors conclude that vibration energy harvesting has potential, but the effective performance depends on the structure and its design. For the long-term, PVDF (polyvinylidene fluoride) may have advantages over PZT in spite of a lower harvesting performance [107]. A review of piezoceramic materials for energy harvesting [105] notes lead-free piezoelectric materials as a perspective choice, with the recent improvement of piezoelectric properties, e.g., BaTiO_3_, for better performance during service compared with PZT.

Most of the published information on such energy harvesters comes from research and development studies. To the best knowledge of the author, no literature on the long-term performance of piezoelectric energy harvesters documenting damage or failures is yet available. Recent developments in energy conversion and storage may change the perspectives for piezoelectric energy harvesting for SHM, but predicting these still remains difficult.

## 5. Discussion and Outlook

The range of effects on piezoelectric SHM-transducers from the operating environment is quite broad. Identifying the causes for deterioration of transducer performance and choosing the appropriate mitigation approach requires periodic or continuous monitoring of the SHM system. An improved and more detailed analysis of such data, e.g., with data fusion and AI, will yield a better understanding of damage mechanisms and their relevant time-scales, providing a basis for the development of specific and cost-efficient mitigation measures. Improving signal-to-noise ratio and easily detecting and eliminating signals from non-relevant sources in the analysis rather than by preset filters before data acquisition may be advantageous in strongly varying operational environments. Miniaturization of electronic hardware and simultaneous software developments have significantly increased the computing power of the SHM systems and thus provided approaches for faster, more detailed and accurate signal analysis. This allows for efficient detection and elimination of non-relevant signals from other sources and for signal de-noising, thus improving the quality of the SHM data. However, miniaturization has also made the hardware more vulnerable and, thus, may reduce the long-term reliability of the equipment. Even if PZT-transducers reliably operate for many years, hardware problems may require more maintenance or even replacement. It is questionable whether state-of-the-art hardware nowadays will reach the service life of, e.g., that of the Voyager spacecraft, still operating after 45 years under the harsh deep space conditions.

As an outlook, a paper by Cawley and coauthors [108] deserves attention. Successful implementation of SHM requires what the authors call “closing the gap” between research and industrial application (cite): “*Reasons for the slow transfer from research to practical application of structural health monitoring include lack of attention to the business case for monitoring, insufficient attention to how the large data flows will be handled and the lack of performance validation on real structures in industrial environments*”. The business case, i.e., cost for SHM versus potential financial loss in case of system failure and the validation on real applications are key aspects. Besides the points summarized in the conclusion in [108], the following factors will likely play a role in future developments toward this goal; PZT currently is the main piezo-ceramic materials used in transducers for SHM with structural waves or vibrations. However, the lead content is problematic for health and environmental reasons and there are plans to ban its use. Therefore, recent research efforts are aimed at the development of lead-free piezo materials with comparable performance. These new developments, independent of which materials will become the main replacement of PZT, first pose the problem that no experience on their long-term service performance is available. Further, many known lead-free piezoelectric materials are less efficient than PZT, especially in sensitivity. In addition, Curie-temperatures of the lead-free piezoelectric materials may be lower than that of PZT. Therefore, the proposed ban of PZT may result in making SHM methods with alternative measurement principles more attractive, both with respect to performance and cost.

## 6. Conclusions

Variations in temperature or elevated temperatures and mechanical vibrations or impact likely constitute the major effects causing degradation of the performance of piezoelectric transducers in long-term SHM monitoring. Placing the transducers in an environment at sufficiently low and, if possible, stable temperature, mitigates the effects to some extent. “Sufficiently low” depends on the Curie-temperature (T_C_), i.e., the transition temperature, of the PZT type of the transducer. The larger the margin between maximum service temperature and T_C,_ the higher the probability is for the safe, long-term operation of transducers. Stable temperature environments will further reduce or even eliminate pyroelectric effects causing spurious signals. There are commercially available piezoelectric transducers operating at elevated and lower temperatures (with ranges specified by the manufacturers). For low temperatures, operational experience is still quite limited. Mitigation of mechanical effects requires suitable mounting devices for the transducers, providing adequate protection of transducers and of the signal transmission and power cables connected to them. Non-contact SHM with either laser- or imaging-based methods are on the way to become a competitor for piezoelectric-based SHM. Development of ever increasing computational power at relatively low cost is one of the drivers. New inspection methods, e.g., employing drones or unmanned aerial vehicles (UAV), increasingly perform periodic SHM and the potential of this technology is not yet fully explored [109]. Drones may be equipped with several complementary NDT methods, also providing coarse first surveys to be followed-up by local inspection with higher resolution. Artificial intelligence methods look promising for fast image analysis with a high probability of detection of (potential) damage. Preventive maintenance, i.e., exchanging SHM system components before the end of their service life, is one mitigation approach that results in the high technical availability of the system. Implementing redundancy, e.g., additional transducers, is another approach. Preventive maintenance will profit from improved predictive models, e.g., digital twins for defining the best time, the least effort, or the lowest cost for such actions. Predictive maintenance is a current research topic and definitely worthwhile to follow. Both approaches, however, imply higher cost. Non-contact inspection methods may provide solutions avoiding several of the problems related to piezoelectric transducers in variable service environments with research in that area also being worthwhile to be followed. In commercial SHM, the performance requirements versus the budget of the client essentially define the “optimal” solution for mitigation of any potential problems, independent of the technology. Designing SHM-systems for easy and cost effective maintenance and repair, as well as implementing self-monitoring, is crucial. Software “life-time” and respective support are issues that also deserve attention for long-term SHM, especially with respect to support and upgrades for commercial codes. New regulatory interventions, e.g., analogous to that requiring lead-free transducers, may also affect SHM services in the future. Making SHM mandatory for critical structures or infrastructure and specifying probabilities of detection for damage may have a significant impact on technology developments and the related cost. Therefore, the perspectives of the different SHM-technologies are difficult to predict in the long-term.

## Data Availability

No new data were created or analyzed in this study.
